# Real-Time Analysis and Visualization for Single-Molecule Based Super-Resolution Microscopy

**DOI:** 10.1371/journal.pone.0062918

**Published:** 2013-04-30

**Authors:** Adel Kechkar, Deepak Nair, Mike Heilemann, Daniel Choquet, Jean-Baptiste Sibarita

**Affiliations:** 1 University of Bordeaux, Interdisciplinary Institute for Neuroscience, Bordeaux, France; 2 CNRS UMR 5297, Bordeaux, France; 3 Goethe-University Frankfurt, Institute of Physical and Theoretical Chemistry, Frankfurt, Germany; Humboldt University, Germany

## Abstract

Accurate multidimensional localization of isolated fluorescent emitters is a time consuming process in single-molecule based super-resolution microscopy. We demonstrate a functional method for real-time reconstruction with automatic feedback control, without compromising the localization accuracy. Compatible with high frame rates of EM-CCD cameras, it relies on a wavelet segmentation algorithm, together with a mix of CPU/GPU implementation. A combination with Gaussian fitting allows direct access to 3D localization. Automatic feedback control ensures optimal molecule density throughout the acquisition process. With this method, we significantly improve the efficiency and feasibility of localization-based super-resolution microscopy.

## Introduction

Single-molecule localization and reconstruction techniques have been instrumental in the recent boom of the application of super-resolution microscopy to answer physiologically relevant questions involving sub-diffraction molecular organization [1]–[3]. Generally, the stochastic optical reconstruction is composed of three steps: (i) the acquisition of several tens of thousands images of single molecules from the sample; (ii) the precise localization of up to a million isolated single emitters and (iii) the visualization of the final super-resolved image reconstructed from the positions of detected individual molecules. The sequential nature of these steps, together with the high acquisition frame rate and the complexity of the processing step, usually prevent the user from viewing the super-resolution images during the acquisition in real time. As a result, the user cannot evaluate the data prior to performing the post-processing, leading to a tremendous loss of time and resources since the overall acquisition pipeline has to be fragmented. Additionally, localization-based super-resolution techniques require an optimal density of molecules to be performed in an optimal way. This can ideally be achieved if it can be measured and adjusted in real-time, involving streaming processing.

Since the emergence of single-molecule based super-resolution microscopy, many efforts have been made to develop new localization algorithms. Knowledge of the Point Spread Function (PSF) is used to find the position and intensity of molecules. PSF engineering, using for example astigmatic lenses, allows for the retrieval of the axial position [4], [5]. Practically, Gaussian fitting of individual fluorescence spots is the most popular localization method since it is the most precise [6]. It is also the most time-consuming method, and the time required to reconstruct the final image remains an obstacle to data production in routine. Recently, various methods were proposed to address the issue of computation time such as RapidSTORM [7] (Neubeck& Van Gool algorithm), QuickPALM [8] (classical Högbom ‘CLEAN’ algorithm), LivePALM [9] (fluoroBancroft algorithm), radial symmetry centers [10] or Maximum Likelihood Estimation (MLE) [11]. Most of these methods are based on massive parallel architecture, using either multiprocessor hardware [7], [8] or graphic processing unit (GPU) [11] for speeding up the localization step. These methods are very efficient in terms of computation time but are either limited to off-line processing, 2D localization or by a relatively slow acquisition rate. Though the compatibility of real-time application is mentioned in the context of various methods, the non-linearity provided by massively parallel processing, combined with the acquisition hardware constrains, doesn’t ensure this rule to be verified until it is effectively implemented on a microscope setup. Indeed, a major constraint of real-time processing is that it requires every single frame to be processed separately in a very short amount of time. It does not permit massively parallel approaches [7], [8], [11], [12] to be performed, since only tens of molecules have to be localized and visualized in only few milliseconds (for example 10 ms at 100 images per second acquisition frame rate). Therefore, the processing time usually exceeds the readout rates of fast EM-CCD cameras. We demonstrate a method for real-time reconstruction with automatic feedback-loop control, without compromising the localization accuracy. Compatible with high frame rates of EM-CCD cameras, it relies on a wavelet segmentation algorithm [13], together with a mix of CPU/GPU (Graphic Processing Unit) implementation. The use of a watershed algorithm allows an efficient localization rate, a key parameter for optimal acquisition and feedback control [14]. A combination with Gaussian fitting enables a direct processing of 3D localization. This step is achieved by NLLS (Non-Linear Least Square) minimization implemented on massively parallel GPU hardware architecture. With a similar philosophy to previous report with MLE iterative minimization [11], each GPU processor computes a single molecule fitting. Real-time feedback control, only possible thank to online processing, allows compensating for molecule density fluctuations, enabling optimal molecule density during the whole acquisition process. Fluctuations, mostly due to bleaching or photo-conversion effects, are inherent to localization methods and reduce their efficiency. We demonstrate that the presented method improves the efficiency and feasibility of single-molecule based super-resolution microscopy for routine biological investigations.

## Materials and Methods

### Immunocytochemistry

COS7 cells were plated on 18 mm coverslips and fixed using 4% paraformaldehyde and sucrose, washed with PBS and with PBS containing 1% BSA. They were incubated with 50 mM NH_4_Cl for 5 minutes prior to permeabilization. Cells were permeabilized using 0.1% Triton and incubated with PBS containing 1% BSA for 30 minutes. They were then incubated with mouse-anti-beta-tubulin antibody (T4026, Clone2.1, Sigma) for 30 minutes and washed several times with PBS containing 1% BSA. The primary antibodies were then revealed by incubating the cells with a secondary antibody, anti-mouse IgG secondary labelled with Alexa Fluor 647 (A21245, Invitrogen), for 30 minutes at room temperature.

### Single-molecule Based Super-resolution Microscopy

Samples were imaged the next day at room temperature in a closed chamber (Ludin Chamber, Life Imaging Services, Switzerland) mounted on an inverted motorized microscope (Nikon Ti, Japan) equipped with a 100×1.45NA PL-APO objective and a perfect focus system, allowing long acquisition in oblique illumination mode (Roper, France). Imaging was performed in an extracellular solution containing reducing and oxygen scavenging system, according to the dSTORM protocol [15]. At the beginning of the experiment, the ensemble fluorescence of Alexa Fluor 647 was first converted in to dark state using a 640 nm laser (Coherent, USA) at 30–50 kW/cm^2^ intensity. Once the ensemble fluorescence was converted into the desired density of single molecules per frame, the laser power was reduced to 7–15 kW/cm^2^ and imaged continuously at 80 FPS for 20,000 frames. The number of single molecules detected per frame was controlled by using a 405 nm laser (Omicron, Germany). The laser powers were adjusted to keep a specific level of stochastically activated molecules which were well separated during the acquisition. Both the ensemble and single molecule fluorescence was collected by the combination of a dichroic and emission filter (D101–R561 and F39–617 respectively, Chroma, USA and quad-band dichroic filter (Di01-R405/488/561/635, Semrock, USA). The fluorescence was collected using a sensitive 512×512 EM-CCD (Evolve, Photometric, USA). 3D localization was performed using the N-STORM astigmatic lens located in front of the CCD camera. The acquisition sequence was driven by Metamorph software (Molecular Devices, USA) in streaming mode at 80 FPS (12 ms exposure time) using an area equal to or less than 256×256 pixel as region of interest. We used multicolour fluorescent microbeads (Tetraspeck, Invitrogen) to register long-term acquisitions and to correct for lateral drifts and chromatic shifts. A spatial resolution of 14 nm was measured using centroid determination on 100 nm Tetraspeck beads acquired with similar signal to noise ratio than single-molecule images. Images were analyzed and reconstructed online using the WaveTracer module integrated into Metamorph software, running on a Intel Xeon E5645@2.4 GHz personal computer (Dell) equipped with a Nvidia Quadro 4000 graphic card.

### Implementation Details

**Figure 1:**
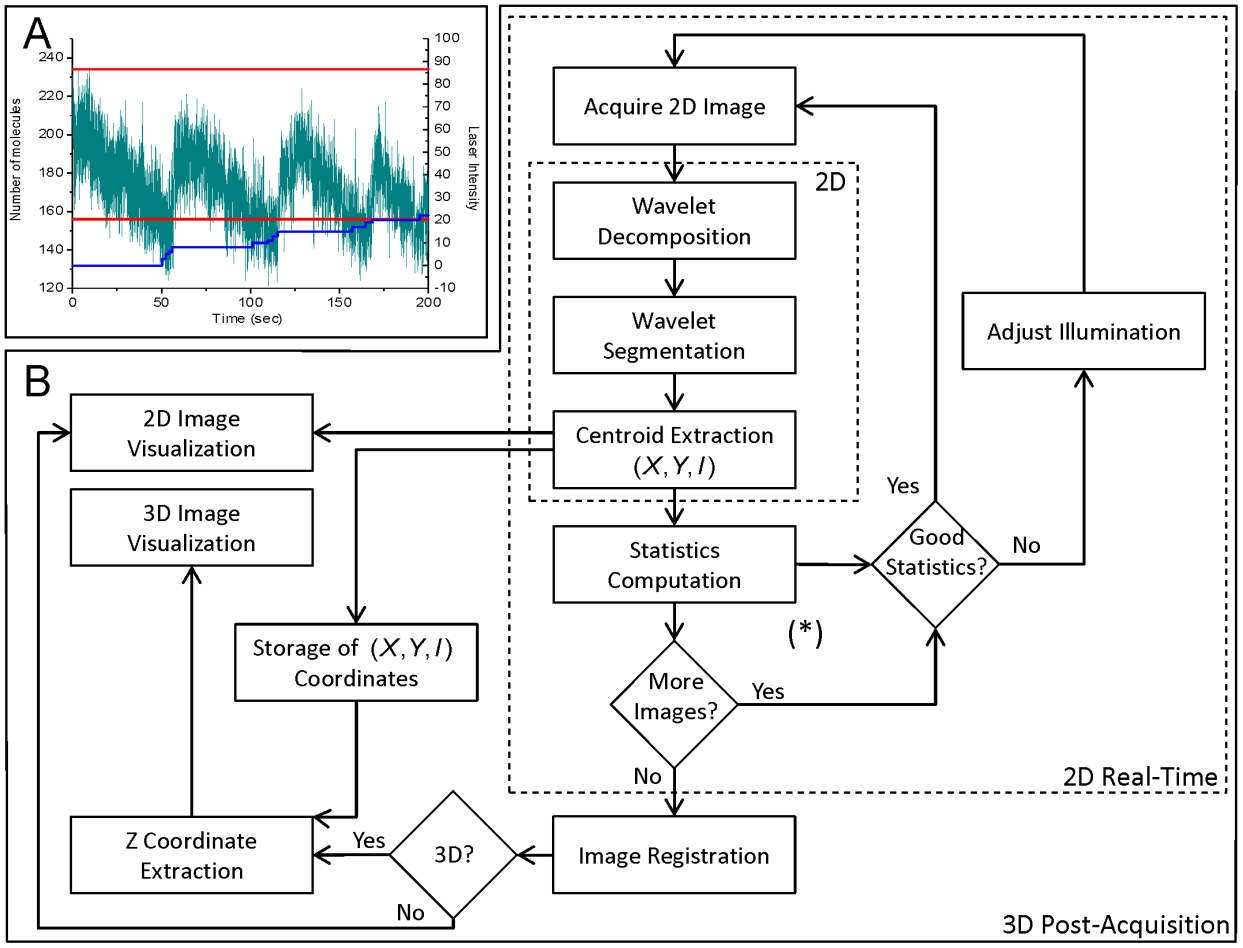
Architecture of WaveTracer software. (**A**) Graph illustrating the automatic real-time control of the number of molecules detected per frame. The number of localization (green line) fluctuates between a set maximum and minimum (red line). It is controlled by a 405 nm laser (blue line). When the number of localized molecules falls outside the minimum and maximum thresholds, the laser power is automatically adjusted to keep the density of molecules ideal for accurate localization. (**B**) Different computation steps for real-time super-resolution reconstruction. The 2D localization algorithm and the visualization of the super-resolved image are performed in real-time with an automatic feed-back control based on the statistic extraction. 3D coordinates extraction is performed at the end of the acquisition. If required, at the end of the acquisition process, the fitting of the preliminary localized molecules is performed. If a fiduciary marker is present, it will be tracked for image registration.

We have implemented an optimized framework for 2D real-time, i.e. streaming, localization and reconstruction, followed, if needed, by a post-acquisition 3D reconstruction. Statistics extracted during real-time localization allow the automatic feedback control on the microscope illumination device, in order to optimize molecule density during the acquisition (**Fig. 1** **.A**). The organizational chart of the method is detailed in **Fig. 1** **.B**. First, the images are analysed in real-time using a wavelet based algorithm [13] which we optimized for speed using a mix of CPU/GPU implementation. If 3D computation is required, positions and intensities of all localized molecules are stored into memory. Second, astigmatism based 3D localization is performed sequentially to the real-time reconstruction by 7×7 anisotropic Gaussian fitting around the stored molecules’ positions. Gaussian fittings are performed in parallel using GPU. The detailed implementation of these 2 steps is described below.

#### a) 2D real-time localization

**Figure 2:**
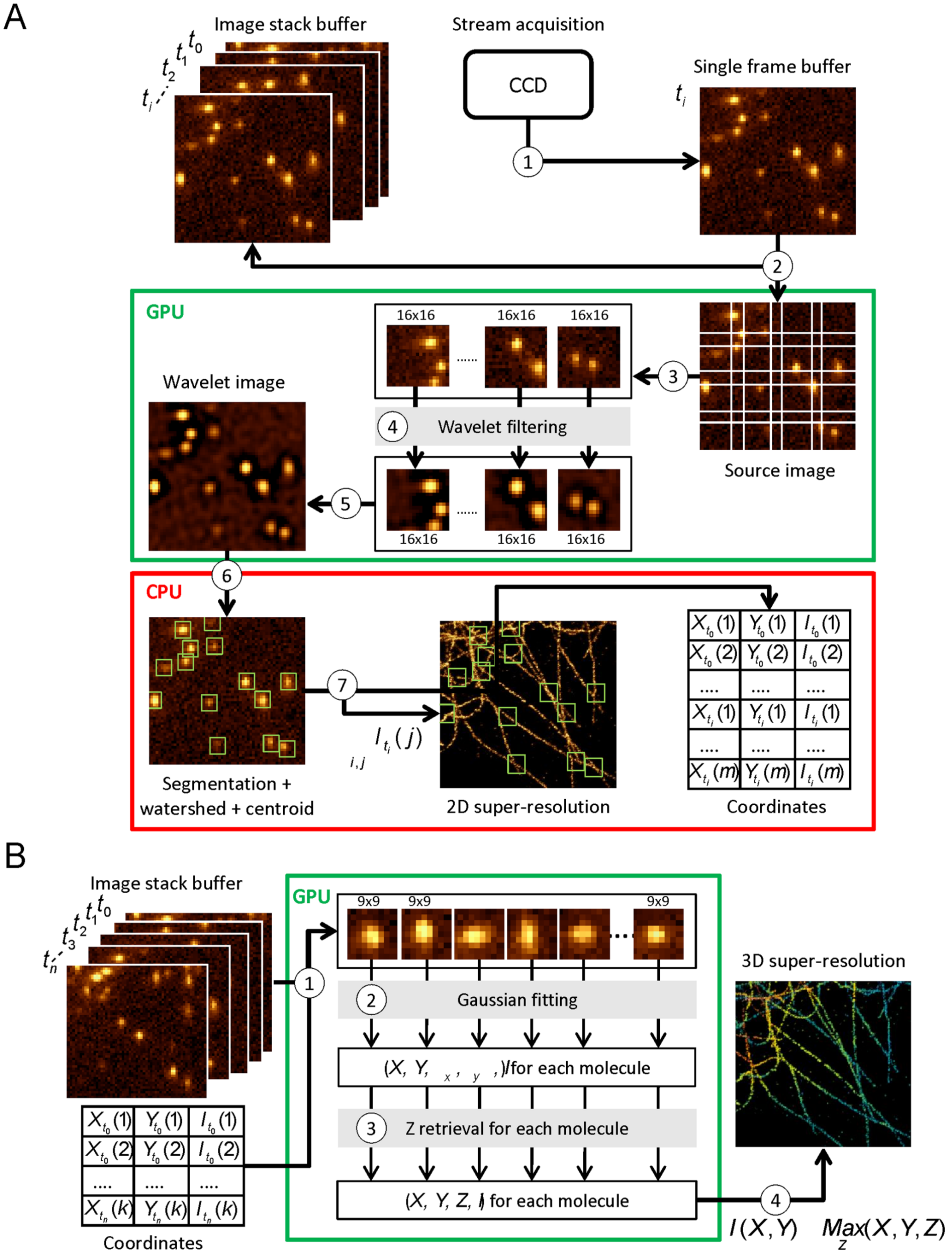
Implementation details of WaveTracer software. (**A**) 2D real-time localization steps: 1) During the acquisition process, images are temporarily transferred to the CCD camera buffer. 2) The current image is transferred to the GPU memory for processing. 3) The image is split into 16×16 overlapping sub-images and sent to different processors of the GPU. 4) Wavelet filtering is performed in parallel on each sub-image. 5) Sub-images are stitched back to reconstruct the filtered image. 6) The filtered image is transferred to the CPU for thresholding, watershed processing and centroid extraction. 7) The super-resolution image is then reconstructed and the localized molecule coordinates are saved into the memory for later 3D analysis. (**B**) 3D post-acquisition localization steps: 1) Images are split into 7×7 sub-images centered on localized molecule coordinates. 2) Anisotropic Gaussian fitting is performed on each sub-image in parallel on GPU. 3) Axial coordinate retrieval of each localized molecule is performed in parallel on GPU. 4) 3D reconstruction is made from the (X,Y,Z) coordinates of all the localized molecules.

During the acquisition process, images are temporarily stored in the CCD camera memory buffer. In streaming mode, each image remains in this buffer during a time period corresponding to the exposure time. After this period of time, it is stored to the computer’s memory and replaced by the next image frame. To perform real-time processing, we only have this short time interval to access and process this image and display the super-resolution reconstruction. We here describe the key implementations of WaveTracer for real-time localization and reconstruction (**Fig. 2** **.A**). First, this frame is transferred to the GPU global memory. Second, the image in the GPU memory space is then split into small 16×16 pixel overlapping regions to be processed in parallel. Third, the wavelet filtering is performed in parallel on each sub-image. Since the fast “à trous” wavelet decomposition algorithm we use is based on multiple 5 pixels line convolutions, each pixel can be computed independently from each other. This filtering step is therefore well suited with the massively parallel architecture of GPU. Fourth, once the parallel filtering is done, the filtered image is stitched back from the resulting individual sub-images, and transferred to the CPU memory. Fifth, a thresholding and a watershed algorithm are performed to identify single molecules and separate molecules in close proximity one to each other. The localization coordinates of each identified molecule are then extracted from their centroid. Finally, the list of coordinates of the localized molecules is stored into the memory, and is used for real-time 2D reconstruction of super-resolution image. If needed, the list of coordinates is used at the end of the acquisition process for post-acquisition 3D reconstruction.

#### b) 3D post-acquisition reconstruction

**Figure 3:**
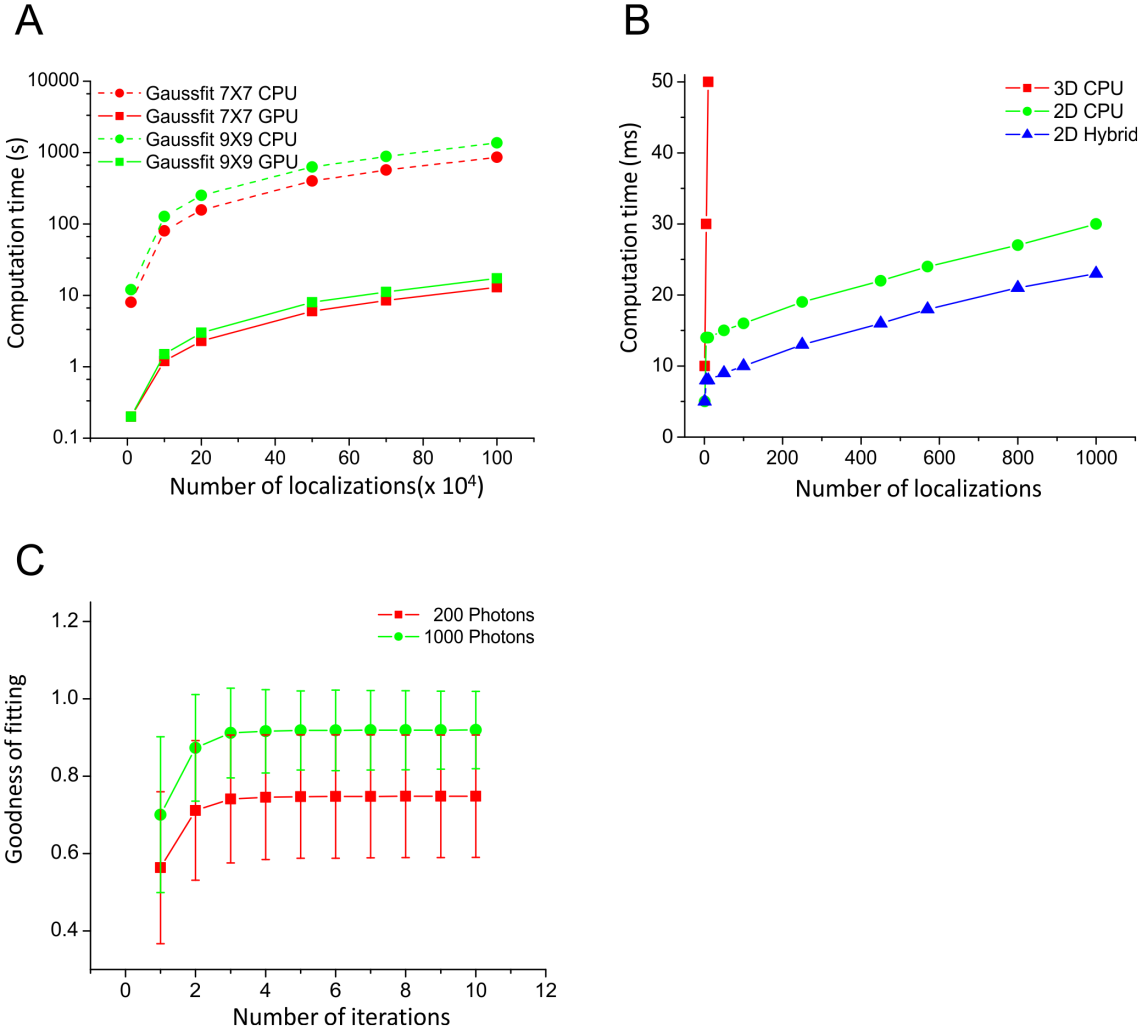
Benchmarking. (**A**) Performance of Gaussian fitting, with (full lines) or without (dashed lines) using GPU, for two different sizes of fitted region. A speed-up factor of about 70 is obtained for the GPU implementation versus the CPU implementation. (**B**) Performance of the localization algorithms in real-time mode. The 2D localization is performed frame by frame in real-time with CPU (green line) and GPU (blue line). Gaussian fitting using CPU (red line) can only process few molecules in 50 ms. Both algorithms are benchmarked on an Intel Xeon E5645@2.4 GHz personal computer equipped with a Nvidia Quadro 4000 graphic card. (**C**) Convergence of the NLLS minimization iterative process for anisotropic Gaussian fitting performed on a 7×7 pixels ROI. Measurements were average from 1,000 molecules, simulated with 200 and 1000 photons per molecule.

For 3D extraction, we perform anisotropic Gaussian fitting of astigmatic single-molecule data followed by *Z* coordinate retrieval, sequentially with the 2D real-time analysis (**Fig. 2** **.B**). In this manner, the level of parallelization offered by GPU is optimal, since it is much more efficient to process simultaneously the large amount of molecules corresponding to the entire acquisition (**Fig. 3** **.A**). This procedure also overcomes the constraints related to the speed of the camera in streaming mode, which prevents real-time Gaussian fitting based computation for fast acquisition frame rates (see **Fig. 3** **.B**). Starting from the acquired images and the list of localization coordinates, the images are divided into small 7×7 pixel regions centered on each coordinates. All the sub-images are transferred to the GPU and processed in parallel by Gaussian fitting, using NLLS minimization, in order to compute the width and height 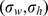 of the PSF. Then, the *Z* coordinate for each molecule is retrieved by mean-square error minimization using the astigmatic calibration curve 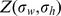 of the optical system. Finally, the 3D super-resolution image is reconstructed from the *(X, Y, Z, I)* list of coordinates. We used a maximum of ten iterations in the NLLS minimization process, corresponding to the convergence plateau (**Fig. 3** **.C**). Even if based on a different minimization method, off-line implementation of Gaussian fitting is similar to the one provided by the previous report [11]. It has the advantage to provide over a million fitting steps in only a few seconds using a standard non-expensive GPU hardware. In combination with our real-time localization pipeline with intensity feedback control, it provides an optimized online localization based super-resolution solution, outperforming tested offline solutions.

#### c) Automatic feedback control

**Figure 4:**
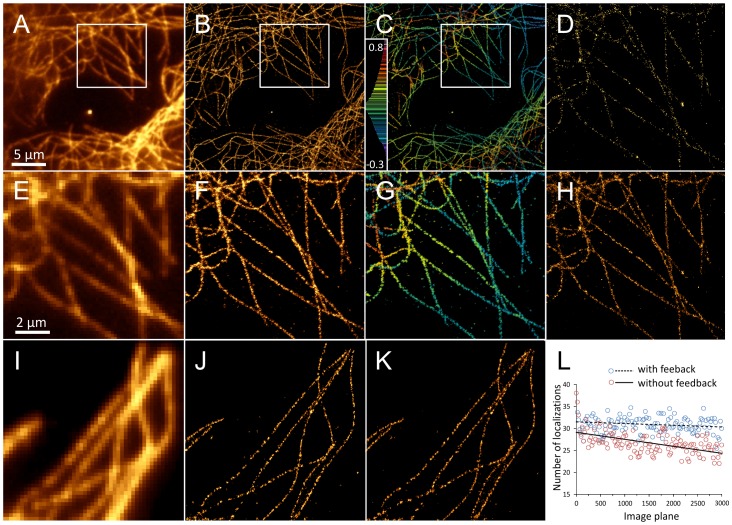
Real-time super-resolution imaging. (**A**) Diffraction-limited epifluorescence image of microtubules labeled with Alexa Fluor 647. (**B**) 2D super-resolved image of the cell in figure (**A**), reconstructed in real-time from 20,000 frames and 1.2 million single-molecule localizations. (**C**) 3D super-resolved image of the microtubules of figure (**A**) obtained only 15 seconds just after the end of the acquisition. Colors encode for the axial position, in µm. (**E**) A selected region of interest (ROI) from figure (**A**). (**F**) A selected ROI from figure (**B**). (**G**) Corresponding ROI from figure (**C**). (**D**), (**H**) Intermediate real-time visualization obtained after 1,000 and 4,000 frames respectively. (**I**) Diffraction limited epifluorescence image of microtubules labeled with Alexa Fluor 647. (**J**), (**K**) 2D super-resolved images of the cell in figure (**I**), reconstructed in real-time from 3,000 frames, without (78,341 localizations) and with (96,298 localizations) feedback control respectively. (**L**) Graph of the number of localizations over the number of images, without (red) and with (green) feedback loop control. Solid and dashed black lines represent their respective trends. For better clarity, only one point over 25 points is displayed.

In order to ensure an optimal molecule density all along the acquisition process, we perform an automatic feedback control on the activation laser power, based on the localization statistics computed in real-time. For each frame, the average and maximal density of molecule localization per 32×32 pixels windows are computed. If during 20 frames, they both pass below or above a threshold set at +/−15% of the initial value, the intensity of the 405 nm laser is adjusted accordingly. The automatic feedback control, possible thanks to the real-time localization capability, allows to optimize the acquisition process. **Fig. 4** **.I-L** illustrate such optimization performed on the first 3,000 frames of a same sample.

### Benchmarking and Comparison with Others Localization Methods

We have benchmarked our method on simulation data, and compared its performance with referenced open-source software like QuickPALM [8] and RapidStorm [7]. They are both based on multithreading implementation which consists in partitioning the program into many tasks that can be processed in parallel, linking its performance to the number of available processors (five for this benchmarking). The GPU based localization software reported previously [11] was not explicitly tested since the available package provides only the fitting step, not the preprocessing step. However, this method was recently compared to RapidStorm [16]. RapidStorm is based on a mixture of levenberg-Marquardt fitter and MLE; QuickPALM relies on the Högbom ‘CLEAN’ algorithm for spot finding, followed by a center of mass algorithm to compute the spot position and shape (i.e. width and height for 3D localization).

For each software package, we have computed the localization accuracy, the localization rates (i.e. the fractions of true positive, false positive and false negative events) as well as the computing speed in offline and real-time mode, in 2D and 3D. The detection rate is the ability of the algorithm to detect individual molecules in a noisy image. Given a total number of simulation particles (*N_S_*), a true positive (*TP*) detection was defined as a molecule present in the analyzed and simulated data set within a radius of one pixel. When a particle present in the simulated data had no matching detection in the analyzed data set within a radius of one pixel, it was counted as a false negative (*FN*) detection. Similarly, a false positive (*FP*) detection was defined as the identification of a molecule in the analyzed data set that was not present in the simulated data within a radius of one pixel. We used ratio, recall and precision, three quantification rates defined as *N_TP_/N_S_* + *N_TP_/(N_TP_+N_FN_)* and *N_TP_/(N_TP_+N_FP_)* respectively.

**Figure 5:**
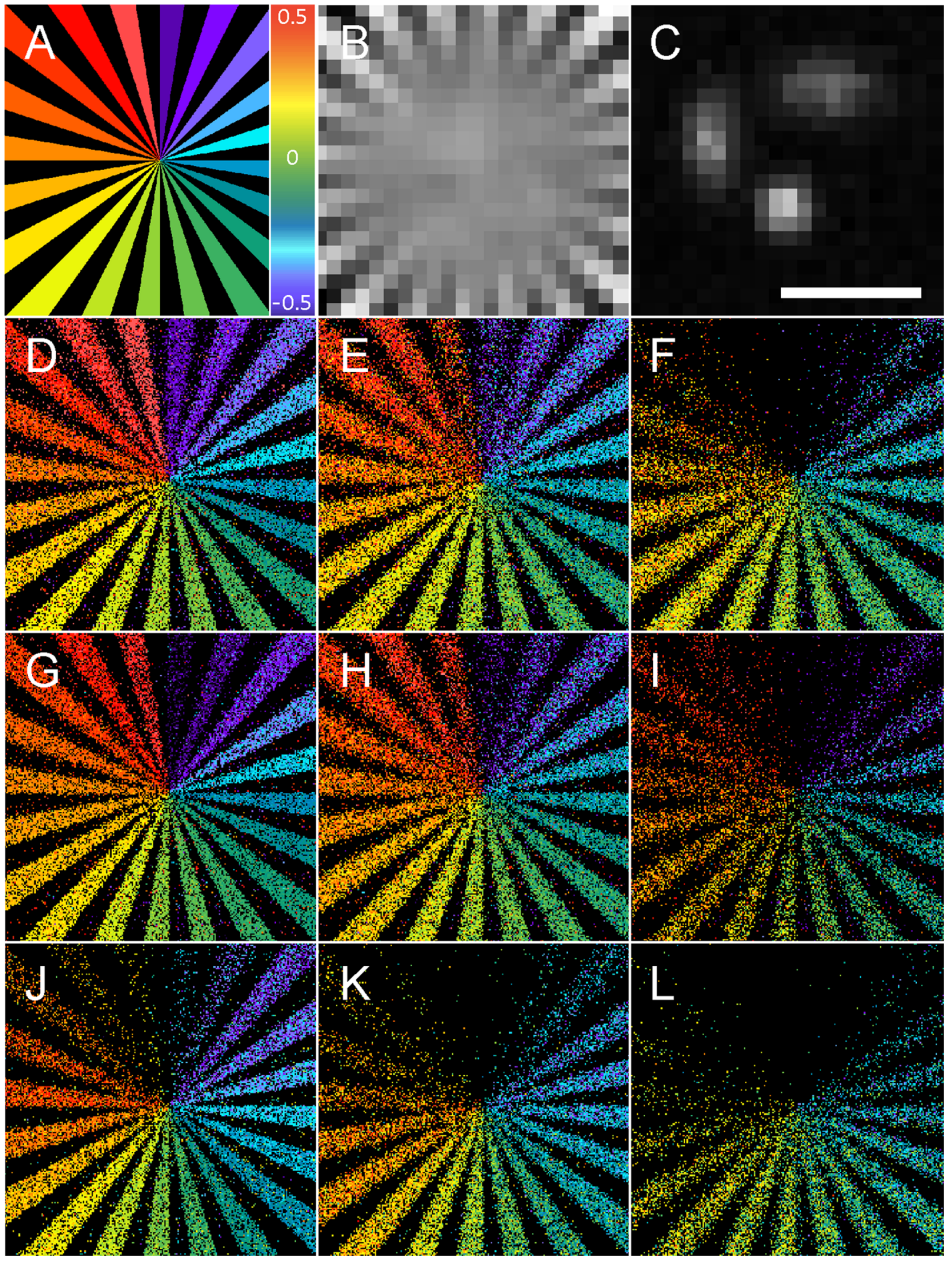
Simulations. (**A**) 3D test patterns made of 40 sunburst alternating black and white stripes, used for single molecule based super-resolution microscopy simulations. The pattern is 1 µm thick, with consecutive stripes distant from 50 nm in the axial direction. (**B**) Diffraction limited image of the test pattern. (**C**) Example of isolated single point emitters located inside the test pattern, convolved with 3D astigmatic point spread function (PSF). (**D–F**) Test pattern reconstruction performed by WaveTracer software for 1000, 200 and 100 photons/molecule respectively. (**G–I**) Test pattern reconstruction performed by RapidSTORM software for 1000, 200 and 100 photons/molecule respectively. (**J–L**) Test pattern reconstruction performed by QuickPALM software for 1000, 200 and 100 photons/molecule respectively. Scale bar is 1 µm.

**Figure 6:**
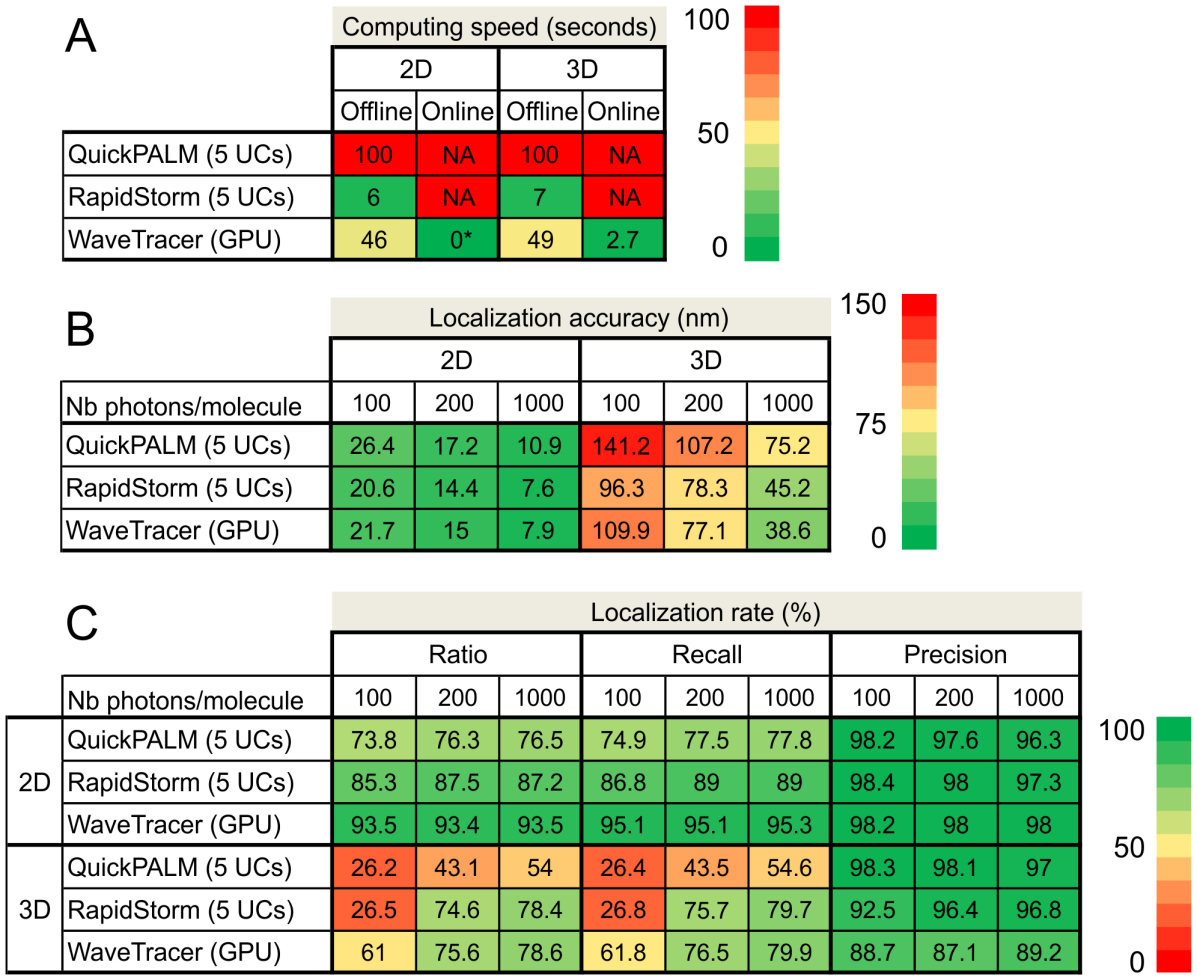
Benchmarking. (**A**) Speed benchmarking for 2D and 3D localization, in offline and real-time modes, and comparison with QuickPALM and RapidSTORM software. Benchmarking was performed on an image stack of 30,000 planes composed of 286,665 molecules. The 0* value mentioned for 2D online means that the localizations are performed in parallel with the acquisition, and that no extra processing time is required. (**B**) Localization accuracy benchmarking in 2D, 3D and for 3 different numbers of photons per molecule, and comparison with QuickPALM and RapidSTORM software. (**C**) Recall and precision detection rates benchmarking for 3 different numbers of photons per molecule, and comparison with QuickPALM and RapidSTORM software.

In order to qualitatively monitor the localization accuracy, we generated 2D and 3D test patterns made of 40 sunburst alternating black and white stripes (**Fig. 5**). For 2D simulations, molecules were randomly positioned within the white stripes. For 3D simulations based on astigmatism, molecules were positioned within the white stripes, each stripe being located at a different Z position between −500 nm to +500 nm around the focal plane. Consecutive stripes are 50 nm apart in the axial direction (**Fig. 5** **.A**). We performed simulations consisting of isolated single point emitters located inside stripes convolved by 2D or 3D point spread function (PSF) with isotropic and anisotropic Gaussian shape respectively. Molecules were randomly distributed with an average density of 0.5 molecule per µm^2^ (**Fig. 5** **.C**). Blurred signal was then sampled on a 64×64 pixelated matrix, with a pixel size of 100 nm providing Nyquist sampling in the visible light range. Finally, digital images were corrupted by a combination of Gaussian and Poisson noise, simulating a limited number of photons and CCD electronic read-out noise. In order to test the localization efficiency of each method for various typical fluorophores, we used 3 different simulation dataset with respectively 100, 200 and 1000 detected photons per molecule per image. For each condition, we generated a stack of 30,000 images composed of 286,665 molecules. These reconstructions allow to illustrate the localization accuracy and rate of each method for various signal to noise ratio (SNR), in 2D and 3D. The corresponding quantitative measurements are summarized in **Fig. 6**.

**Fig. 6A** shows a detailed comparison of the computational speed of the three methods. In offline mode, our method took more time than RapidSTORM, since our 2D localization was performed frame by frame for real-time purpose. We didn’t optimize our 2D localization method using massively parallel architecture, since our main target was real-time localization (i.e. no delay after the acquisition process). We can localize about 150 molecules within 10 ms (**Fig. 3** **.B**), which is sufficient for streaming processing with rapid EM-CCD camera. In the case of 3D real-time, 2D localization is performed in real-time and 3D localization offline. WaveTracer takes full advantage of GPU capability and could outperform tested methods like RapidSTORM, with more than 80,000 fits per second. In terms of localization accuracy (**Fig. 6B**), the three methods show similar performances with an advantage for WaveTracer and RapidSTORM in comparison to QuickPALM, as illustrated in **Fig. 5**. Similarly, the detection rates (**Fig. 6C**) are similar for all three methods, with a slight advantage of WaveTracer in the capability to localize true positives (ratio and recall rates) even in low SNR conditions, an advantage provided by the wavelet segmentation.

## Results and Discussion

Here, we illustrate a new advanced analysis method, named WaveTracer, which enables optimized real-time data reconstruction for single-molecule super-resolution microscopy. Spatial coordinates of each localized molecule are retrieved in real-time in two or three dimensions, down to the few tens of nm resolution. Automatic feedback control on the activation laser power is performed based on real-time localization statistics, allowing the regulation of the optimal density during the acquisition process (**Fig. 1**). For 2D real-time localization, we use a wavelet-based segmentation algorithm, a method which outperforms the Gaussian fitting (MLE or NLLS) in terms of speed, while maintaining similar localization accuracy [13]. Wavelet filtering allows to get rid of most of the image noise and background, enabling rapid and accurate object segmentation. A watershed algorithm is applied after segmentation to allow close molecules to be separated. In order to respect the constraint of real-time analysis, i.e. the analysis of each image in streaming, desirable for feedback control, we opted for a hybrid CPU/GPU implementation (**Fig. 2**). The use of graphic processors is primarily to speed up the wavelet decomposition (**Fig. 2** **.A**). Since the wavelet decomposition is very similar to a convolution, the processing of each pixel of the image is independent of each other. The local nature of wavelet decomposition suits perfectly the parallel pixel-by-pixel processing on a GPU. Practically, the computation time is about two times faster compared to a classical CPU implementation (**Fig. 3** **.B**). With our method, we reached to process and reconstruct the super-resolution image from single-molecule data acquired at any EM-CCD frame rate in real-time. This enables the computation of the localization density along the acquisition, and provides the capability to perform a laser intensity feedback control to optimize the single molecule density. In addition, fiduciary markers that are present in the field of view are automatically tracked using a nearest-neighbour algorithm, allowing real-time image registration.

For 3D localization based on astigmatism [4], [5], we combined wavelet segmentation with Gaussian fitting. The PSF is elliptically shaped above and below the focal plane with a shape factor and dimension changing along the optical axis. After calibrating the optical system, the axial coordinate of a localized molecule can then be retrieved by performing a local fitting of the raw data around the coordinates computed by the wavelet segmentation process, followed by a mean-square-error (MSE) minimization with the calibration function. A Gaussian fitting of a 7×7 pixel area allows computing the ellipse parameters necessary to retrieve the position and length of the small and large axes. Since this fitting step is time consuming when computed on a CPU, we performed a GPU implementation. Nevertheless, real-time constrains do not allow massively parallel implementation. Consequently, even a GPU implementation does not allow for Gaussian fitting to be performed in real-time (**Fig. 3B**). This is because the GPU is more adapted to large data set computation, where the configuration time is negligible compared to the processing time (**Fig. 3A**). Therefore, we implemented the following 2 steps for 3D reconstruction (**Fig. 1**): i) compute the 2D localization with intensity feedback control in real-time using wavelet segmentation; ii) compute the fitting and 3D extraction sequentially, right after the end of the acquisition. The fitting of different spots being independent of each other, the GPU implementation can efficiently do the treatment in parallel (**Fig. 2** **.B**). We implemented the NLLS fitting method in GPU using Levenberg-Marquardt optimization, since it offers faster processing with similar accuracy compared to MLE algorithm [17]. Thanks to the molecule coordinates and intensities computed during the 2D real-time localization step, the Gaussian fitting converged rapidly after only few iterations (**Fig. 3** **.C**). Therefore, we used a maximum of 10 iterations per molecule as a convergence stopping criteria. The fitting of a million molecules is almost 70 times faster in the case of GPU versus CPU (**Fig. 3** **.A**). GPU implementation enables computing the axial coordinates of 1.2 million molecules in less than 15 seconds, compared to about 15 minutes in the case of a CPU. This enables the user to access the 3D information just a few seconds right after the acquisition process, which can be assimilated to real-time compared to the acquisition time. Thanks to this unique combination, we could achieve more than 80,000 fits per second with optimal molecule density, outperforming other tested reference methods and keeping similar localization accuracy.

We have illustrated the performance of our algorithm on experimental dSTORM [15] data recorded from COS7 cells with microtubules stained with Alexa Fluor 647 (**Fig. 4**), as well as on 2D and 3D synthetic patterns (**Fig. 5**). On experimental data, we observe the microtubule organization at different planes with an average lateral and axial resolution of 20 nm and 50 nm, respectively. 2D super-resolution images were provided in real-time during the streaming acquisition at 80 FPS for a 200×200 pixel image. An average number of 60+/−20 localizations per image was kept constant during the whole acquisition process by adjusting the irradiation power accordingly. The 3D reconstruction of 1.2 million molecules was obtained less than 15 seconds just after the acquisition. Simulated test pattern reconstructions allowed us to benchmark, in offline mode, the computation speed and the localization accuracy and rate of WaveTracer with popular free software RapidSTORM and QuickPALM (**Fig. 6**).

Our method allows overcoming two of the major limitations in single-molecule based super-resolution microscopy, which are the computation time required to process the massive amount of data (up to 0.5 Gigabyte per minute) and the regulation and control of the optimal molecule density. Using a regular computer, it offers access to 2D and 3D information during the acquisition, without compromising the resolution. Real-time analysis saves the user from performing post-processing, which dramatically slows down the overall acquisition pipeline. In addition, it allows optimizing the acquisition parameters by feedback control on the microscope’s illumination system. If desired, only the molecule localizations and super-resolution images can be stored and manipulated, saving time, disk space and bandwidth. These are key features if we want to consider using such methodology in high throughput context to screen molecule organization at high spatial resolution. We thus think that the proposed approach will further promote the use of one of the most popular and powerful super-resolution imaging method of today.
